# Serum lipids are associated with nonalcoholic fatty liver disease: a pilot case-control study in Mexico

**DOI:** 10.1186/s12944-021-01526-5

**Published:** 2021-10-10

**Authors:** Yvonne N. Flores, Aryana T. Amoon, Baolong Su, Rafael Velazquez-Cruz, Paula Ramírez-Palacios, Jorge Salmerón, Berenice Rivera-Paredez, Janet S. Sinsheimer, Aldons J. Lusis, Adriana Huertas-Vazquez, Sammy Saab, Beth A. Glenn, Folasade P. May, Kevin J. Williams, Roshan Bastani, Steven J. Bensinger

**Affiliations:** 1 Department of Health Policy and Management, Fielding School of Public Health, University of California, Los Angeles (UCLA), Los Angeles, CA USA; 2 UCLA Center for Cancer Prevention and Control and UCLA-Kaiser Permanente Center for Health Equity, Fielding School of Public Health and Jonsson Comprehensive Cancer Center, Los Angeles, CA USA; 3 Unidad de Investigación Epidemiológica y en Servicios de Salud, Morelos, Instituto Mexicano del Seguro Social, Cuernavaca, Morelos Mexico; 4 UCLA Lipidomics Laboratory, David Geffen School of Medicine, Los Angeles, CA USA; 5 Laboratorio de Genómica del Metabolismo Óseo, Instituto Nacional de Medicina Genómica (INMEGEN), Ciudad de México, Mexico; 6 Centro de Investigación en Políticas, Población y Salud, Universidad Nacional Autónoma de México, Ciudad de México, Mexico; 7 UCLA Department of Human Genetics and Computational Medicine, Los Angeles, CA USA; 8 Department of Biostatistics, UCLA Fielding School of Public Health, Los Angeles, CA USA; 9 UCLA Department of Medicine, Division of Cardiology, David Geffen School of Medicine, Los Angeles, CA USA; 10 UCLA Department of Microbiology, Immunology & Molecular Genetics, David Geffen School of Medicine, Los Angeles, CA USA; 11 UCLA Department of Medicine, Vatche and Tamar Manoukian Division of Digestive Diseases, David Geffen School of Medicine, Los Angeles, CA USA; 12 Pfleger Liver Institute, UCLA David Geffen School of Medicine, Los Angeles, CA USA; 13 Department of Medicine, VA Greater Los Angeles Healthcare System, Los Angeles, CA USA; 14 UCLA Department of Biological Chemistry, David Geffen School of Medicine, Los Angeles, CA USA

**Keywords:** Lipidomics, Triacylglycerol desaturation, Triglycerides, Nonalcoholic fatty liver disease, NAFLD, Biomarkers, Latinos, Mexican, Cross-sectional study

## Abstract

**Background:**

Nonalcoholic fatty liver disease (NAFLD) is a leading cause of chronic liver disease and cirrhosis. NAFLD is mediated by changes in lipid metabolism and known risk factors include obesity, metabolic syndrome, and diabetes. The aim of this study was to better understand differences in the lipid composition of individuals with NAFLD compared to controls, by performing direct infusion lipidomics on serum biospecimens from a cohort study of adults in Mexico.

**Methods:**

A nested case-control study was conducted with a sample of 98 NAFLD cases and 100 healthy controls who are participating in an on-going, longitudinal study in Mexico. NAFLD cases were clinically confirmed using elevated liver enzyme tests and liver ultrasound or liver ultrasound elastography, after excluding alcohol abuse, and 100 controls were identified as having at least two consecutive normal alanine aminotransferase (ALT) and aspartate aminotransferase (AST) (< 40 U/L) results in a 6-month period, and a normal liver ultrasound elastography result in January 2018. Samples were analyzed on the Sciex Lipidyzer Platform and quantified with normalization to serum volume. As many as 1100 lipid species can be identified using the Lipidyzer targeted multiple-reaction monitoring list. The association between serum lipids and NAFLD was investigated using analysis of covariance, random forest analysis, and by generating receiver operator characteristic (ROC) curves.

**Results:**

NAFLD cases had differences in total amounts of serum cholesterol esters, lysophosphatidylcholines, sphingomyelins, and triacylglycerols (TAGs), however, other lipid subclasses were similar to controls. Analysis of individual TAG species revealed increased incorporation of saturated fatty acyl tails in serum of NAFLD cases. After adjusting for age, sex, body mass index, and *PNPLA3* genotype, a combined panel of ten lipids predicted case or control status better than an area under the ROC curve of 0.83.

**Conclusions:**

These preliminary results indicate that the serum lipidome differs in patients with NAFLD, compared to healthy controls, and suggest that assessing the desaturation state of TAGs or a specific lipid panel may be useful clinical tools for the diagnosis of NAFLD.

**Supplementary Information:**

The online version contains supplementary material available at 10.1186/s12944-021-01526-5.

## Background

Nonalcoholic fatty liver disease (NAFLD) is a leading cause of chronic liver disease with an estimated global prevalence of 24% [1]. NAFLD is the accumulation of excess fat in the liver, without a clear secondary cause of lipid accumulation (e.g.*,* significant alcohol consumption, use of steatogenic medication, or genetic disorders) [2]. NAFLD ranges from simple hepatic steatosis to nonalcoholic steatohepatitis (NASH) characterized by hepatic inflammation and hepatocellular injury. NAFLD and NASH can progress to cirrhosis and hepatocellular carcinoma (HCC) [3, 4]. Treatment options for NAFLD are limited and it has become the fastest growing cause of HCC among liver transplant candidates [5]. Predicting which individuals will progress from NAFLD to more advanced liver disease remains difficult to assess, and finding prognostic markers remains an important objective. Therefore, the identification of key biomarkers that could improve the non-invasive detection of NAFLD and the development of new treatment strategies that reduce chronic liver disease incidence and mortality would be a major benefit to public health.

In the United States, NAFLD and NASH are most prevalent among Latinos [6–10], and those of Mexican origin have the highest prevalence [11, 12]. Known risk factors for NAFLD and disease progression include obesity, insulin resistance, metabolic syndrome, and diabetes. NAFLD is found in 80–90% of obese adults, 30–74% of patients with diabetes, and 90% of patients with hyper-lipidemia [6, 13]. Other risk factors include larger waist circumference [14, 15], and older age [16–19], and elevated triglycerides [14, 15, 20–22]. Hypertriglyceridemia has been identified as an important and early predictor of NAFLD [23, 24]. While total cholesterol, often measured as part of a standard lipid panel, is not generally related to NAFLD overall, but cholesterol carrying lipoproteins, including high density, low density, non-high density and very-low density lipoproteins have been associated with NAFLD [25–30].

There is also some evidence that NAFLD may be a heritable disease, in which gene-environment interactions contribute to the progress and severity of disease [31]. A single variant in the *PNPLA3* gene (rs738409) has the most robust and consistent association with hepatic steatosis [32, 33]. This variant is a cytosine to guanine substitution that modifies codon 148 from isoleucine to methionine (I148M). *PNPLA3* encodes a 481 amino acid protein of unknown function that belongs to the patatin-like phospholipase family [34]. The *PNPLA3*-I148M allele is more common among Latinos (49%), than non-Hispanic Whites (23%), or non-Hispanic Blacks (17%) [34–36]. Studies with health workers in Mexico also confirm that the *PNPLA3*-I148M allele is associated with a greater risk of persistently elevated aminotransferase levels [37, 38].

Dysregulation of hepatic lipid metabolism underlies the pathogenesis of NAFLD and alterations in systemic lipid metabolism are found in patients with NAFLD and NASH [3, 39–41]. Various lipid markers have also been associated with NAFLD and NASH including triglyceride [e.g., TAG (48:0)], phosphatidylethanolamine [e.g., PE (40:6)], and lysophosphatidylcholine [e.g., LPC (16:0)] [42]. The very long chain dihydroceramides and polyunsaturated PEs can discriminate NAFLD from NASH subjects [43]. Certain lipid subclasses are generally higher in NAFLD cases, including diacylglycerols [25, 26, 44, 45] and free fatty acids [26, 44], while other lipid subclasses are mostly decreased in NAFLD cases compared to controls (e.g. LPC, [46] PE [25, 46], ceramides (CER) [47], cholesterol esters (CE) [47], and phosphatidylcholines (PC) [25]. However, none of these studies included Latino adults, so very little is understood about how specific lipid types contribute to NAFLD susceptibility among Latinos.

The primary objective of this pilot study was to better understand differences in the lipid composition of individuals with NAFLD compared to healthy controls, and to identify potential detection markers for NAFLD in an understudied population that is at high-risk for developing NAFLD. Targeted direct infusion “shotgun” lipidomics were performed on serum biospecimens from 98 NAFLD cases and 100 controls in Mexico, to observe changes in the circulating lipidome of patients with NAFLD. The study hypothesis was that the circulating lipids in serum among NAFLD cases would be different than in healthy controls. A secondary objective of this study was to investigate how individual triacylglycerol (TAG) species may vary between NAFLD cases and controls.

## Methods

### Study design

The study used a nested case-control design and examined lipidomics, genetic, and clinical data to compare a sample of NAFLD cases to healthy controls from adult participants in a longitudinal study in Mexico. All study procedures were approved by the Institutional Review Boards, and all participants provided informed consent. This research followed the Strengthening the Reporting of Observational Studies in Epidemiology (STROBE) guidelines [48].

### Study sample

The Health Worker Cohort Study (HWCS) is investigating the genetic and lifestyle risk factors associated with various chronic diseases in Mexico. The study design and methodology are described in detail elsewhere [49–51]. At baseline (2004–2006) and follow-up (2011–2013 and 2017–2018), study participants completed self-reported questionnaires, a physical examination, and provided blood samples for laboratory testing. A convenience sample of 98 NAFLD cases and 100 healthy controls aged 36 to 78 years, who are long-term participants in the HWCS was used for the current pilot study. The inclusion criteria for this study was having a hepatologist-confirmed diagnosis of NAFLD for the cases, and a history of normal ALT and AST results (< 40 U/L) plus a normal ultrasound elastography result for the controls. Liver enzymes (ALT, AST), total cholesterol, high-density lipoprotein (HDL-C), low-density lipoprotein cholesterol (LDL-C), triglycerides, glucose, body mass index (BMI), waist circumference, and blood pressure were measured. Participants reported fasting for ≥12 h at blood draw. Serum samples that were collected during 2011–2013 and stored at − 70 °C, as well as self-reported data and laboratory results that were obtained during the same time period, were used for the cases and controls. Targeted genetic studies have also been performed in a larger sample of the HWCS participants to identify single nucleotide polymorphisms (SNPs) associated with NAFLD (*n* = 632), osteoporosis (*n* = 689) and other complex diseases (*n* = 1936) [37, 38, 52, 53].

### Clinical confirmation of NAFLD cases and controls

The primary outcome variable was a diagnosis of NAFLD, which was determined by a hepatologist using established guidelines [2]. Liver ultrasound or ultrasound elastrography was used to assess the presence of hepatic steatosis, as well as persistently elevated ALT or AST levels ≥40 international units per litre (IU/L). Additionally, NAFLD cases were confirmed to have no significant alcohol consumption (< 20 g/day for females, < 30 g/per day for males), no competing etiologies for hepatic steatosis (e.g. chemotherapy or toxic exposures), and no coexisting causes of chronic liver disease (e.g. infection with hepatitis B or hepatitis C). Stratified randomization was used to select age- and sex-matched controls from the HWCS participants who had at least two consecutive normal ALT and AST measures (< 40 U/L) over a period of 6 months, during 2004–2006, 2011–2013, and 2017–2018. All controls were confirmed not to have a NAFLD diagnosis from the results of an ultrasound elastrography evaluation that was conducted in January 2018.

### Covariates

#### Body mass index (BMI)

The study participants’ BMI was determined from standardized measures of weight and height and was calculated as a ratio of weight (in kilograms) to height (in square meters). Participants were classified as normal weight (18.5–24.9 kg/m^2^), overweight (25.0–29.9 kg/m^2^) or obese (≥30 kg/m^2^), based on recommendations from the National Heart, Lung, and Blood Institute [54].

#### Diabetes

Type 2 diabetes was defined as having any of the following: a medical history of diabetes (excluding during pregnancy), currently taking medication for diabetes, a plasma glucose level > 125 mg/dL after fasting 12 h, or a random glucose test > 200 mg/dL [55].

#### Metabolic syndrome

Based on the 2009 Criteria for Clinical Diagnosis of the Metabolic Syndrome by Alberti et al., participants were classified as having metabolic syndrome if they presented three or more of the following: (1) waist circumference ≥ 90 cm for males and ≥ 80 cm for females; (2) HDL-C < 40 mg/dL for males and < 50 mg/dL for females; (3) triglycerides ≥150 mg/dL; (4) fasting glucose ≥110 mg/dL, or currently taking medication for elevated glucose; and (5) systolic blood pressure ≥ 130 mmHg, diastolic blood pressure ≥ 85 mmHg, or currently taking medications for high blood pressure [56].

#### *PNPLA3* (rs738409) genotyping

All participants were genotyped for the rs738409 I148M variant using TaqMan assays (Applied Biosystems, Foster City, CA, USA). No discordant genotypes were observed in 50 duplicate samples. The CC genotype corresponds to normal/no risk, the CG represents a medium risk, and the GG genotype corresponds to a high risk. Deviation from Hardy–Weinberg equilibrium was not observed.

### Extraction procedures

Serum samples from 98 NAFLD cases and 100 controls with complete data were identified for lipid extraction. First, samples were thawed and 25ul of serum were pipetted into a glass tube for extraction. A modified Bligh and Dyer extraction [57] was carried out on the serum samples. Prior to biphasic extraction, the Lipidyzer Internal Standard Mix that contains 54 lipid standards across 13 subclasses, was added to each sample (AB Sciex, 5,040,156). The 13 subclasses include: cholesterol esters (CE), ceramides (CER), diacylglycerols (DAG), dihydroceramides (DCER), free fatty acids (FFA), hexosylceramides (HCER), lactosylceramides (LCER), lysophosphatidylcholines (LPC), lysophosphatidylethanolamines (LPE), phosphatidylcholines (PC), phosphatidylethanolamines (PE), sphingomyelins (SM), and triacylglycerols (TAGs). Following two successive extractions, pooling/concentrating of the organic layers were dried down in a Genevac EZ-2 Elite for direct infusion lipidomic analysis. Lipid samples were resuspended in 1:1 methanol/dichloromethane with 10 mM Ammonium Acetate and transferred to robovials (Thermo 10,800,107) for analysis. Samples were processed between November 2018 and February 2019 at the UCLA Lipidomics Laboratory.

### Mass spectrometry lipid analysis

Samples were analyzed on the Sciex Lipidyzer Platform (Framingham, MA, USA) and quantified with normalization to serum volume. This platform has been described elsewhere [58]; briefly, it is a direct infusion-tandem mass spectrometry system utilizing differential mobility. Analysis is carried out over two infusions, with each acquiring roughly half of the lipid targets within the assay. As many as 1100 targeted lipid species can be identified using the Lipidyzer targeted multiple-reaction monitoring (MRM) list, but only 622 were quantified in at least 33% of cases or 33% of controls. The cut point of 33% was chosen to avoid correlational artifact weighted by lipids detected in very few samples. This allowed us to examine nearly 57% of the 1100 targeted lipid species in a sample that was less likely to have missing data. A total of 352 lipids had no missing data in both cases or controls (i.e. were quantified for all 198 samples tested), of which 251 were TAGs. Concentrations were reported as nmoles/mL. Lipid class totals were calculated in each sample by summing the concentration of all individual lipid species within each class and presented in units of nanomoles/mL.

### Statistical analyses

SAS version 9.3 (Cary, NC, USA), Python 3.7 (Fredericksburg, Virginia, USA), R 4.0.0 (Vienna, Austria), and ClustVis [59] were used to conduct the data analyses. Lipids were tested for normal distribution and log-transformed when necessary for specific analyses. To be included in the primary analyses, lipid species had to be measured in at least 33% of either cases or controls. This was done to ensure that lipids with exceedingly low abundance across samples were not included. The median and 25–75% interquartile range (IQR) of specific lipid subclasses and species were determined and presented in box plots with whiskers representing 1.5 times the IQR bounded by the highest and lowest samples. Prior to analysis, outliers with greater than 3 standard deviations (SD) from the mean were excluded from analysis. Two-tailed unpaired Student’s t test were used to determine differences between cases and controls. NAFLD cases and healthy controls were also compared using analysis of covariance (ANCOVA), adjusting for several combinations of covariates, including: age, sex, diabetes status, metabolic syndrome status, BMI status, and *PNPLA3* genotype. Six models were created to adjust for these covariates. Results were visualized using Manhattan plots. False discovery rate (FDR) was computed using the Benjamini-Hochberg method to adjust for multiple comparisons [60]. Pearson’s linear correlation coefficients were calculated for each pair of lipids and presented in a heat map.

Random forest analysis (RFA) was performed on untransformed data to identify lipids with the most impact on case/control status using Python package scikit-learn (Fredericksburg, Virginia, USA), [61]. RFA is a classification technique using thousands of decision trees (a.k.a. a “forest”). Each tree is created based on a subset of samples that are used to predict the remaining samples. Classification is determined by computing frequencies of predictions for each group over the entire forest. To determine the impact of each lipid, impurity-based feature importance was computed (also known as the Gini importance or Mean Decrease in Impurity). The importance of a lipid is computed as the total reduction of the criterion brought by that feature. Larger values suggest a greater influence of that specific lipid. A panel of the 10 most important lipids was generated to determine their sensitivity and specificity to predict NAFLD using receiver operative characteristic (ROC) curves.

## Results

### Study population

Table 1 reports some demographic and basic clinical characteristics of the study sample. Nearly three fourths were female (74%) with a mean age of 61 (SD ±10) years, and a significantly higher proportion of female controls than cases. NAFLD cases had higher levels of AST, ALT, glucose, and TAGs compared to healthy controls. NAFLD cases also had significantly greater rates of diabetes and metabolic syndrome. Although mean BMI was higher in the NAFLD group, a greater proportion of controls were obese (41% vs. 35%). No differences were observed in age, total cholesterol levels, or *PNPLA3* genotype.

**Table 1:** Demographic and clinical characteristics of NAFLD cases and healthy controls (*n* = 198)^a^

	Total	Cases ***n*** = 98	Controls ***n*** = 100	***P***-value^**b**^
**Sex**				
Females	74.8	63.3	86.0	
Males	25.2	36.7	14.0	0.0002
**Age,** years				
Mean (95% CI)	60.7 (59.3–62.2)	59.9 (57.8–62.0)	61.5 (59.5–63.6)	0.2768
37–54	25.3	27.6	23.0	
55–69	49.0	48.0	50.0	
70–78	24.8	24.5	27.0	0.7523
**Biomeasures,** mean (95% CI)				
AST, IU/L	33.8 (31.2–36.4)	42.1 (37.7–46.4)	25.7 (23.8–27.5)	< 0.0001
ALT, IU/L	38.0 (34.4–41.6)	49.8 (43.9–55.8)	26.5 (24.0–28.9)	< 0.0001
Glucose, mg/dL	116.5 (110.2–122.8)	130.5 (119.0–142.0)	102.8 (98.8–106.7)	< 0.0001
Triglycerides, mg/dL	173.4 (157.4–189.4)	197.7 (168.6–226.9)	149.5 (137.0–162.0)	0.0031
Cholesterol, mg/dL	196.5 (190.6–202.4)	192.4 (184.1–200.6)	200.5 (191.9–209.1)	0.1751
***PNPLA3*** **Genotype**				
CC	14.1	14.1	14.0	
CG	46.2	40.4	52.0	
GG	39.7	45.5	34.0	0.2131
**BMI,** kg/m^2^				
Mean (95% CI)	28.3 (27.6–29.0)	29.9 (28.9–31.0)	26.7 (25.9–27.6)	< 0.0001
Normal	28.8	18.4	39.0	
Overweight	33.3	46.9	20.0	
Obese	37.9	34.7	41.0	< 0.0001
**Diabetes**				
No	73.2	60.2	86.0	
Yes	26.8	39.8	14.0	< 0.0001
**Metabolic Syndrome**				
No	41.4	31.6	51.0	
Yes	58.6	68.4	49.0	0.0057

^a^ Results are presented as %, unless otherwise stated.

^b^*P*-value results of chi-square tests (categorical) and t-tests (continuous)

Abbreviations: *NAFLD* nonalcoholic fatty liver disease, *CI* confidence interval, *AST* aspartate aminotransferase, *ALT* alanine aminotransferase, *BMI* body mass index

### Alterations in serum TAGs, LPCs, and CEs among NAFLD cases

**Fig. 1:**
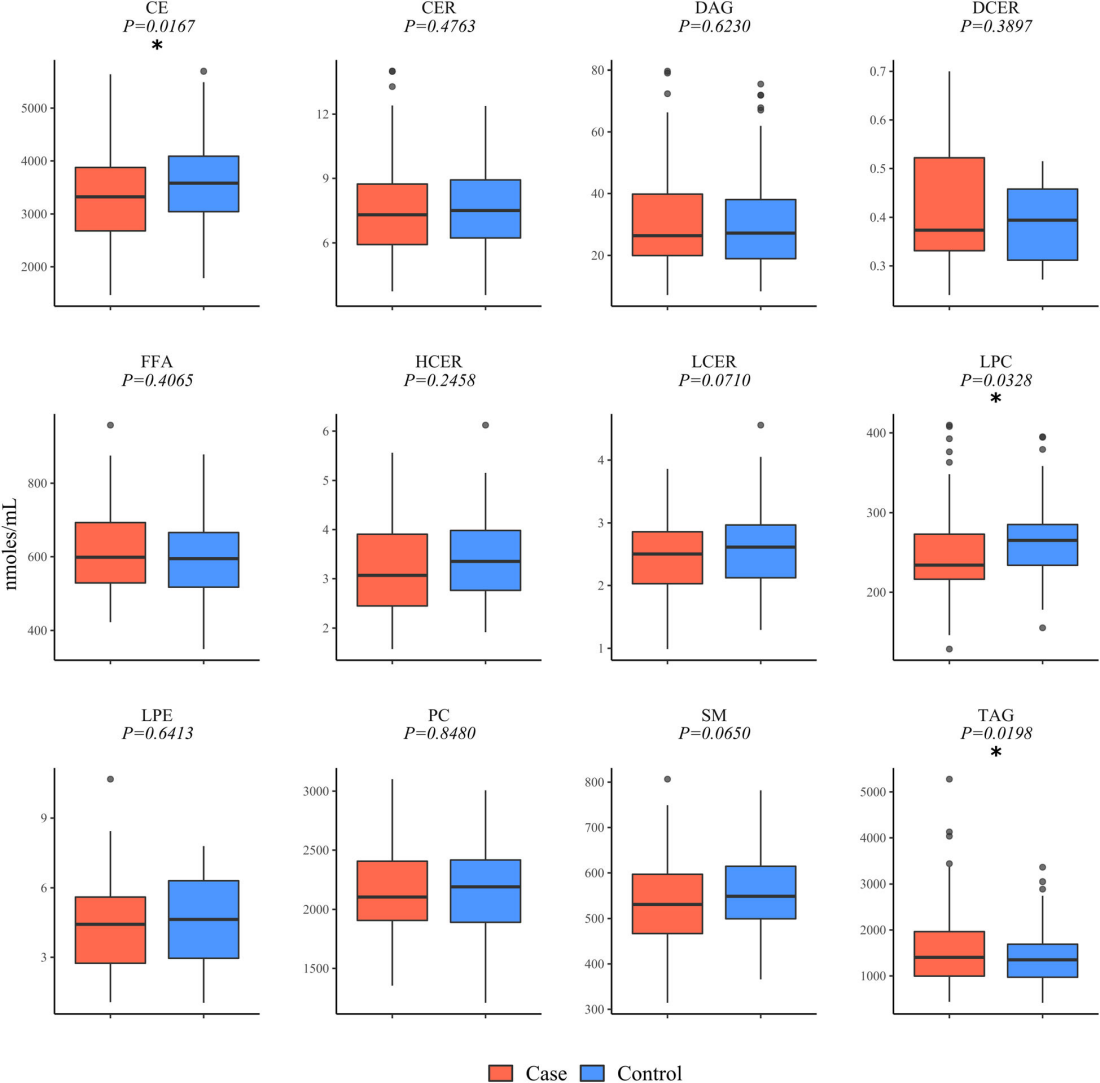
Alterations in serum TAGs, LPCs, and CEs in NAFLD cases *P* values indicated above compare cases and controls (two-tailed unpaired Student’s t test). * *P* value < 0.05. Abbreviations: *CE* cholesterol ester, *CER* ceramide, *DAG* diacylglycerol, *DCER* dihydroceramide, *FFA* free fatty acid, *HCER* hexosylceramide, *LCER* lactosylceramide, *LPC* lysophosphatidylcholine, *LPE* lysophosphatidylethanolamine, *PC* phosphatidylcholine, *SM* sphingomyelin, *TAG* triacylglycerol

An average of 576 individual lipid species were measured from a total of 729 individual lipids detected in the serum of NAFLD cases and controls from 13 distinct subclasses or classes within glycerophospholipid, glycerolipid, sphingolipid, cholesterol, and fatty acids classes [62, 63]. One sample was excluded from analysis for technical reasons. Analysis of subclass totals revealed a significant increase in the total amounts of TAG (*P* = 0.0198), and significant decreases in total CE (*P* = 0.0167) and LPC (*P* = 0.0328) pools (Fig. 1).

For case and control groups (*n* = 98, *n* = 100), the median and 25–75% interquartile range (IQR) are presented in box plots with whiskers representing 1.5x the IQR bounded by the highest and lowest samples. Outliers with greater than 3 standard deviations (SD) from the mean were excluded from analysis.

### Correlations between lipid species

Pearson correlations were performed between each pair of lipid species (Additional file 1). As expected, within the same subclass, there were strong positive correlations between lipid species across the subclasses, especially among TAGs, DAGs, FFAs, HCERs, and SMs. Between subclasses, many TAGs were strongly correlated positively with DAGs as well as a handful of PCs and CEs. CEs and CERs were also positively correlated with most LPCs, PCs, and SMs, in addition to one another. Additionally, PCs were positively correlated with LPCs and SMs. In the negative direction, several CEs were strongly correlated with DAGs, FFAs, and TAGs. DAGs and HCERs or SMs also shared mostly negative correlations, as did HCERs and LCERs with TAGs. Total concentration of TAGs measured by mass spectrometry was positively correlated with the standard bloodwork clinical measurement of TAGs (Pearson correlation = 0.85). No significant differences were found in total PC, PE, lyso-PE, DAG, SM, CER, LCER, HCER, DCER, and FFA (Additional file 1).

Despite changes in the total pool sizes of CE, LPC, and TAG in the serum of NAFLD cases, a heat map analysis of individual lipid species failed to reveal any discernable pattern of enrichment or losses (Additional files 2 and 3). Thus, it can be concluded that changes in CE, LPC, and TAG pools result from a general change across many individual lipids within these subclasses.

### Enrichment of saturated TAGs in serum of NAFLD cases

**Fig. 2:**
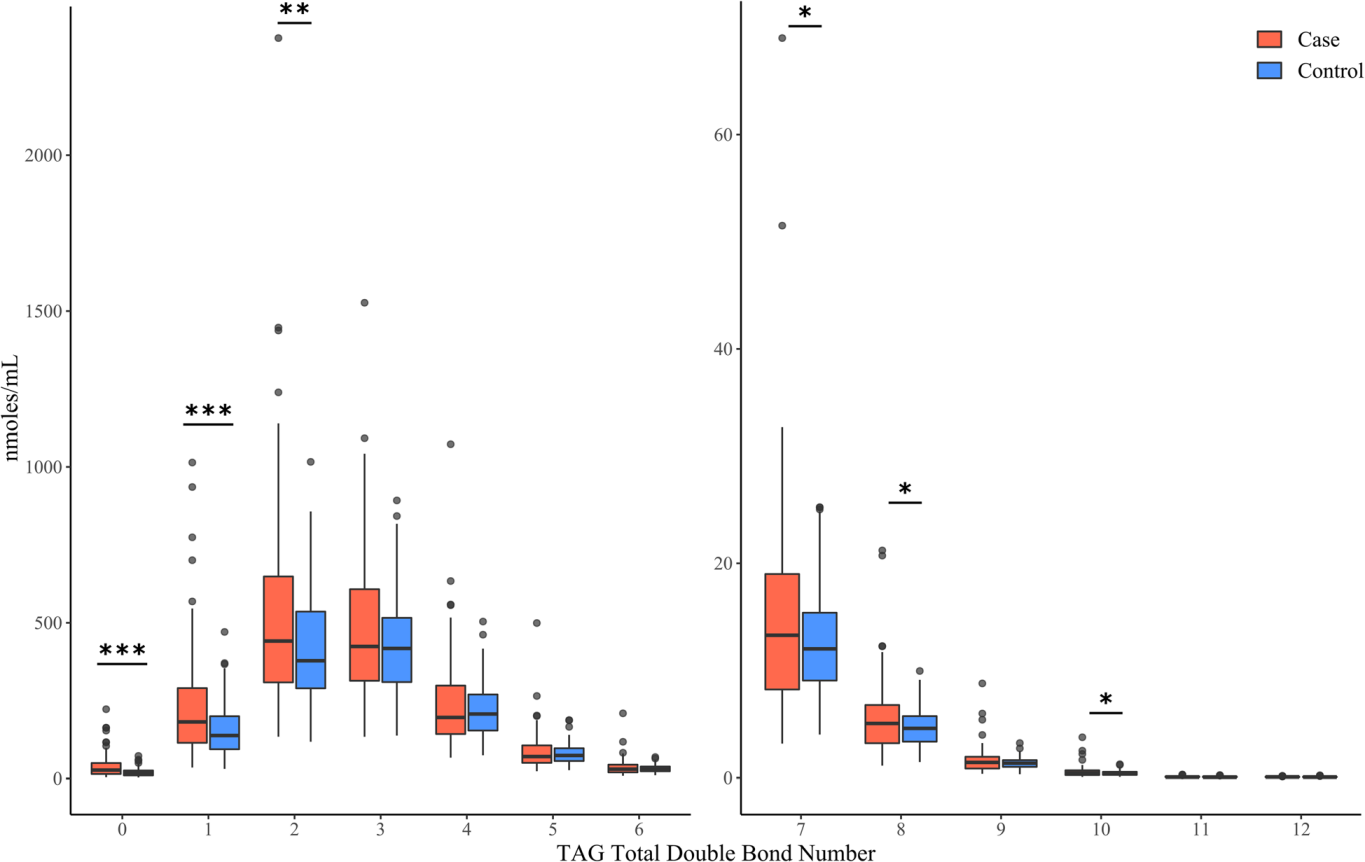
Enrichment of saturated TAGs in serum of NAFLD cases *P < 0.05, **P < 0.01; ***P < 0.001; (two-tailed unpaired Student’s t test). Abbreviations: *NAFLD* nonalcoholic fatty liver disease, *TAG* triacylglycerols

The overall desaturation state of the TAG pool was also examined by summing all TAG species according to the total double bond number. For example, TAGs have three acyl tails that contain variable numbers of double bonds, and summation of these double bound numbers can reveal general information regarding the desaturation state of that lipid subclass. Individual TAG species were binned according to bulk (total) double bond number within a given sample. The concentrations of species with a given double bond number were summed and divided by three to account for the sampling of each acyl tail within the TAG species. Using this approach, we found that TAGs from NAFLD cases were preferentially enriched for acyl tails that were fully saturated or contain few double bonds (Fig. 2). Interestingly, this preferential incorporation of saturated acyl tails was not observed in other subclasses of lipids (e.g., PC or DAGs), indicating a level of specificity for this shift in desaturation state.

For double bond number 0–12, the case and control groups were compared using box and whisker plots that depict the median, 25–75% interquartile range (IQR) and 1.5x the IQR bounded by the highest and lowest samples. Outliers with greater than 3 standard deviations from the mean were excluded from analysis (Fig. 2).

### Overall lipid subclass concentrations between NAFLD cases and controls

Analysis of covariance (ANCOVA) was used to compare the overall lipid subclass concentrations between the NAFLD cases and healthy controls, adjusting for appropriate covariates such as age, sex, BMI, diabetes, metabolic syndrome status, and *PNPLA3* genotype. An individual lipid species was required to be present in at least 33% of either case or control samples for inclusion in these additional studies. This inclusion criteria resulted in two lipid subclasses (DCER and PE) being excluded from further analysis. Similar to the aforementioned results, CE levels were observed to be significantly lower in NAFLD cases in all six models (Table 2; *P* < 0.05). LPCs and SMs were also lower in serum from NAFLD cases in two of the models (*P* < 0.05), after additionally adjusting for age, sex, and *PNPLA3* genotype. Other lipid subclasses were variably enriched or decreased but failed to show statistical significance when adjusted for specific confounders. Of note, we found that TAGs were increased in NAFLD cases (Fig. 1), however, this increase was not significant after adjusting for confounders (Table 2).

**Table 2:** Comparison of overall lipid subclass concentrations between NAFLD cases and healthy controls^*^

Lipid class	Fold^**a**^	***P***-value					
		Model 1^**b**^	Model 2^**c**^	Model 3^**d**^	Model 4^**e**^	Model 5^**f**^	Model 6^**g**^
CE	0.941	**< 0.01**	**< 0.01**	**< 0.01**	**< 0.01**	**< 0.01**	**< 0.01**
CER	1.503	0.19	0.11	0.12	0.47	0.35	0.08
DAG	1.174	0.26	0.23	0.95	0.43	0.35	0.94
FFA	1.013	0.26	0.24	0.63	0.43	0.38	0.62
HCER	1.168	0.64	0.66	0.95	0.86	0.88	0.94
LCER	0.739	0.97	0.97	0.95	0.86	0.88	0.12
LPC	0.960	**0.01**	**0.03**	0.09	0.06	0.14	0.94
LPE	0.972	0.94	0.97	0.84	0.98	0.88	0.94
PC	0.990	0.97	0.97	0.95	0.86	0.88	0.20
SM	0.975	**0.03**	**0.03**	0.18	0.06	0.10	0.94
TAG	1.372	0.19	0.20	0.95	0.24	0.26	0.81

* Results presented are corrected for False Discovery Rate

^a^ Quotient of sum of all log-transformed lipid values in cases divided by sum of all log-transformed lipid values in controls

^b^ Adjusted for age and sex

^c^ Adjusted for age, sex, and *PNPLA3* genotype

^d^ Adjusted for age, sex, diabetes, and metabolic syndrome status

^e^ Adjusted for age, sex, and BMI category

^f^ Adjusted for age, sex, BMI category, and *PNPLA3* genotype

^g^ Adjusted for age, sex, diabetes, metabolic syndrome status, and *PNPLA3* genotype

Abbreviations: *NAFLD* nonalcoholic fatty liver disease, *CE* cholesterol ester, *CER* ceramide, *DAG* diacylglycerol, *FFA* free fatty acid, *HCER* hexosylceramide, *LCER* lactosylceramide, *LPC* lysophosphatidylcholine, *LPE* lysophosphatidylethanolamine, *PC* phosphatidylcholine, *SM* sphingomyelin, *TAG* triacylglycerol

### TAG double bond number concentrations in NAFLD cases and healthy controls

The relative saturation or desaturation state of the acyl tails in the serum TAG pools between the NAFLD cases and healthy controls was also compared. Increases for saturated or single double bond TAGs were observed in nearly all models tested. (Table 3). Thus, measuring the fully saturated TAG pool size may provide additional information that could be of value regarding clinical status of NAFLD patients.

**Table 3:** Comparison of TAG double bond number concentrations in NAFLD cases and healthy controls

# of double bonds	***P***-value					
	Model 1^**a**^	Model 2^**b**^	Model 3^**c**^	Model 4^**d**^	Model 5^**e**^	Model 6^**f**^
0	< 0.1	< 0.01	0.06	< 0.01	< 0.01	0.04
1	< 0.1	< 0.01	0.15	< 0.01	< 0.01	0.12
2	0.05	0.06	0.69	0.06	0.07	0.71
3	0.20	0.28	0.86	0.16	0.24	0.71
4	0.32	0.48	0.83	0.19	0.32	0.62
5	0.26	0.39	0.94	0.12	0.21	0.83
6	0.14	0.21	0.63	0.05	0.09	0.81
7	0.08	0.13	0.42	0.03	0.05	0.55
8	0.10	0.14	0.38	0.04	0.07	0.50
9	0.26	0.36	0.71	0.15	0.22	0.85
10	0.10	0.16	0.37	0.05	0.10	0.53
11	0.45	0.55	0.37	0.49	0.61	0.45
12	0.97	0.90	0.83	0.70	0.83	0.77

^a^ Adjusted for age and sex

^b^ Adjusted for age, sex, and *PNPLA3* genotype

^c^ Adjusted for age, sex, diabetes, and metabolic syndrome status

^d^ Adjusted for age, sex, and BMI category

^e^ Adjusted for age, sex, *PNPLA3* genotype and BMI category

^f^ Adjusted for age, sex, diabetes, metabolic syndrome status, and *PNPLA3* genotype Abbreviations: *NAFLD* nonalcoholic fatty liver disease, *TAG* triacylglycerol

### Difference in individual lipid species between NAFLD cases and controls

**Fig. 3:**
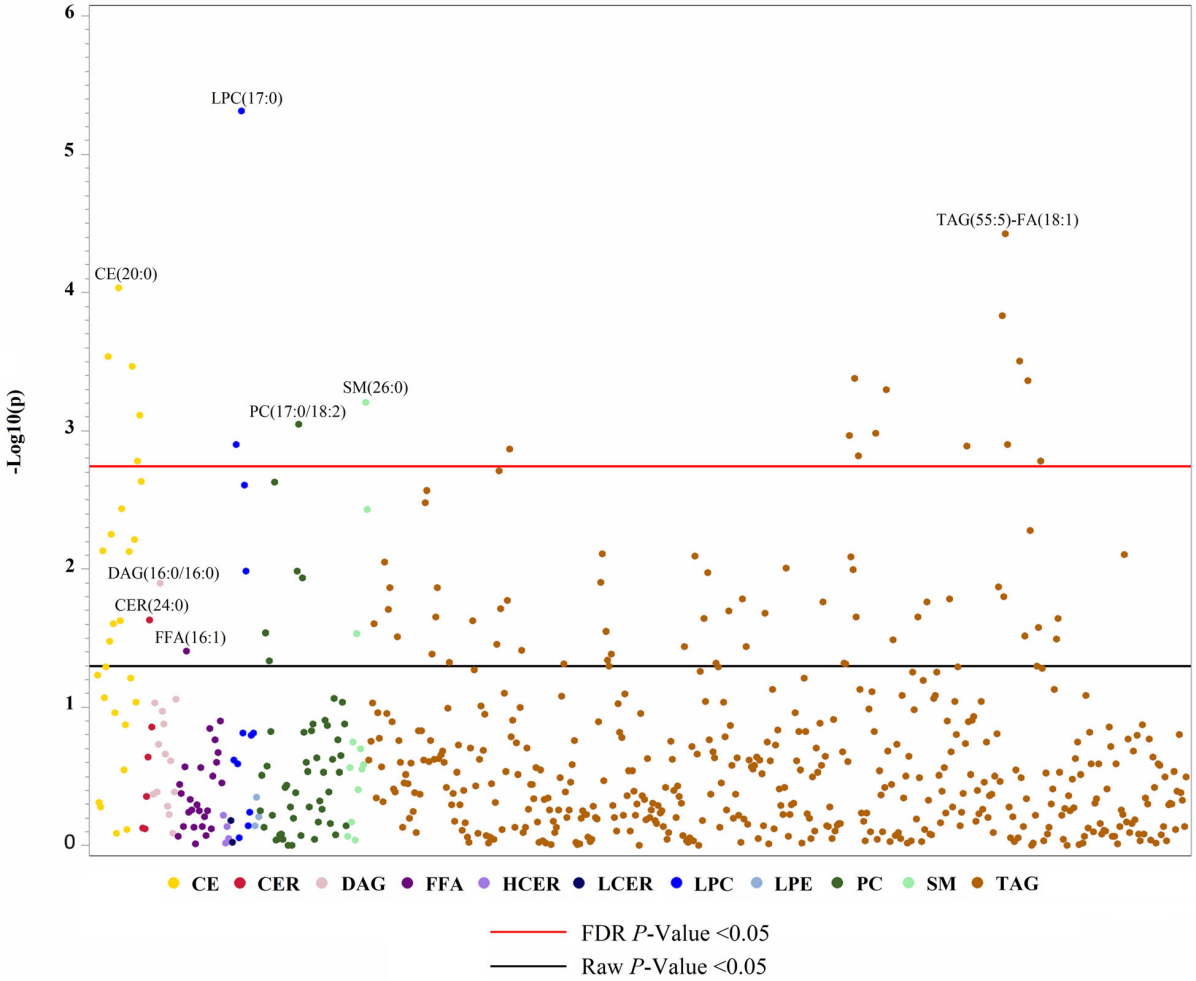
Manhattan plot of difference in individual lipid species between NAFLD cases and controls Select individual lipids of interest are labelled. Lipid classes are color coded as indicated. Abbreviations: *CE* cholesterol ester, *CER* ceramide, *DAG* diacylglycerol, *FFA* free fatty acid, *HCER* hexosylceramide, *LCER* lactosylceramide, *LPC* lysophosphatidylcholine, *LPE* lysophosphatidylethanolamine, *PC* phosphatidylcholine, *SM* sphingomyelin, TAG triacylglycerol

Figure 3 presents a Manhattan plot of the individual lipid species that were observed to be different in NAFLD cases and controls. After adjusting for age, sex, diabetes, metabolic syndrome status, and *PNPLA3* genotype, a total of 94 lipids were significantly different between cases and controls (*P* < 0.05, lipids presented above black bar). Of these lipids, 37 were decreased in NAFLD cases and 57 were increased. After further correction for false discovery rate (FDR), 22 lipids remained significant, including 13 TAGs, of which nine were lower in NAFLD cases compared to controls (*P* < 0.05, lipids presented above red bar). All remaining non-TAG lipids were also decreased in cases compared to controls.

Negative log10 of *P*-values are plotted. Lipid species that were significantly different between cases and controls after adjusting for age, sex, diabetes, metabolic syndrome status, and *PNPLA3* genotype are presented above the black line (*P* < 0.05). Lipid species that remained significant after False Discovery Rate (FDR) correction are presented above the red line (*P* < 0.05) (Fig. 3).

### Random forest analysis to identify key individual lipid species

**Fig. 4:**
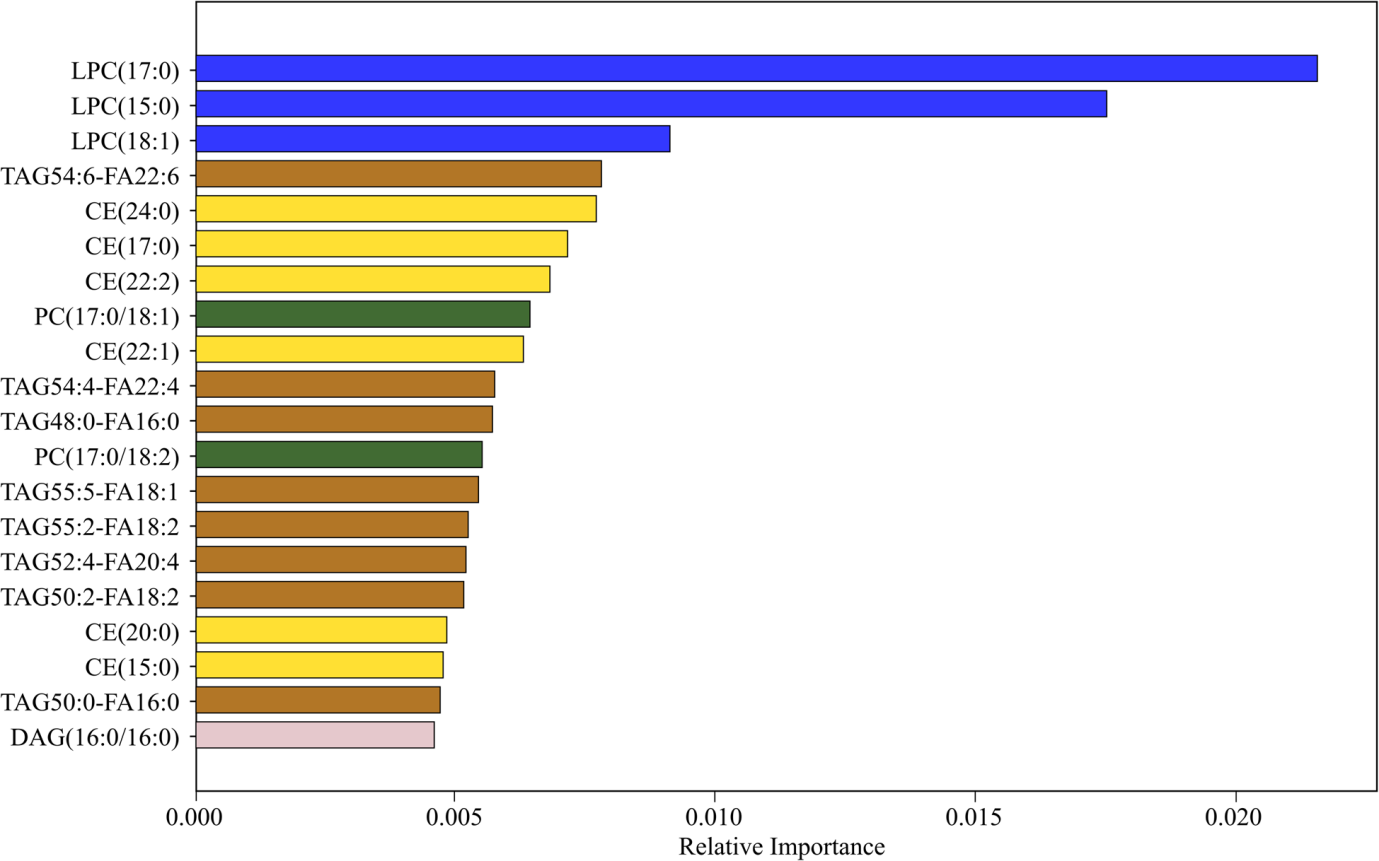
Most important lipids as determined by random forest analysis Gini importance values for indicated lipids are plotted. Although this process can produce slightly different results each time due to the random nature, LPC(17:0), LPC(15:0), and LPC(18:1) were identified as the top three lipids in all random forest analyses. Abbreviations: *LPC* lysohphosphatidylcholine, *TAG* triacylglycerol, *CE* cholesterol ester, *PC* phosphatidylcholine, *DAG* diacylglycerol

A random forest analysis was performed using individual lipid amounts detected in cases and controls. Important individual lipid species consisted of LPCs, TAGs, PCs, and CEs, and a single DAG, with LPC 17:0 ranking as the most important (Fig. 4). Several of the lipid species were in agreement with the results presented in Fig. 3, including CE (20:0), LPC (17:0), PC (17.0/18.2), and TAG (55:5-FA18:1).

Inspection of lipid species identified in the random forest analysis revealed an enrichment for complex lipids containing odd chain saturated acyl tails, suggesting that these specific lipids may provide additional information for identifying NAFLD cases. However, after conducting an area under the receiver operating characteristic curve (AUROC) analysis the of top 20 lipids as determined by random forest analysis, adjusting for age, sex, diabetes, metabolic syndrome, and *PNPLA3* genotype, no single lipid was able to predict NAFLD status better than 71% (Additional file 4).

Of the top 20 lipids identified in the random forest analysis, the following were significantly lower in NAFLD cases than controls: LPC(17:0), LPC(15:0), LPC(18:1), CE(24:0), CE(17:0), CE(22:2), PC(17:0/18:1), CE(22:1), PC(17:0/18:2), TAG55:5-FA18:1, CE(20:0), CE(15:0). While these lipid species were significantly higher in cases than controls: TAG54:6-FA22:6, TAG54:4-FA22:4, TAG48:0-FA16:0, TAG55:2-FA18:2, TAG52:4-FA20:4, TAG50:2-FA18:2, TAG50:0-FA16:0, DAG(16:0/16:0) (Additional file 5).

### AUROC analysis of the top 10 lipids to differentiate cases from controls

**Fig. 5:**
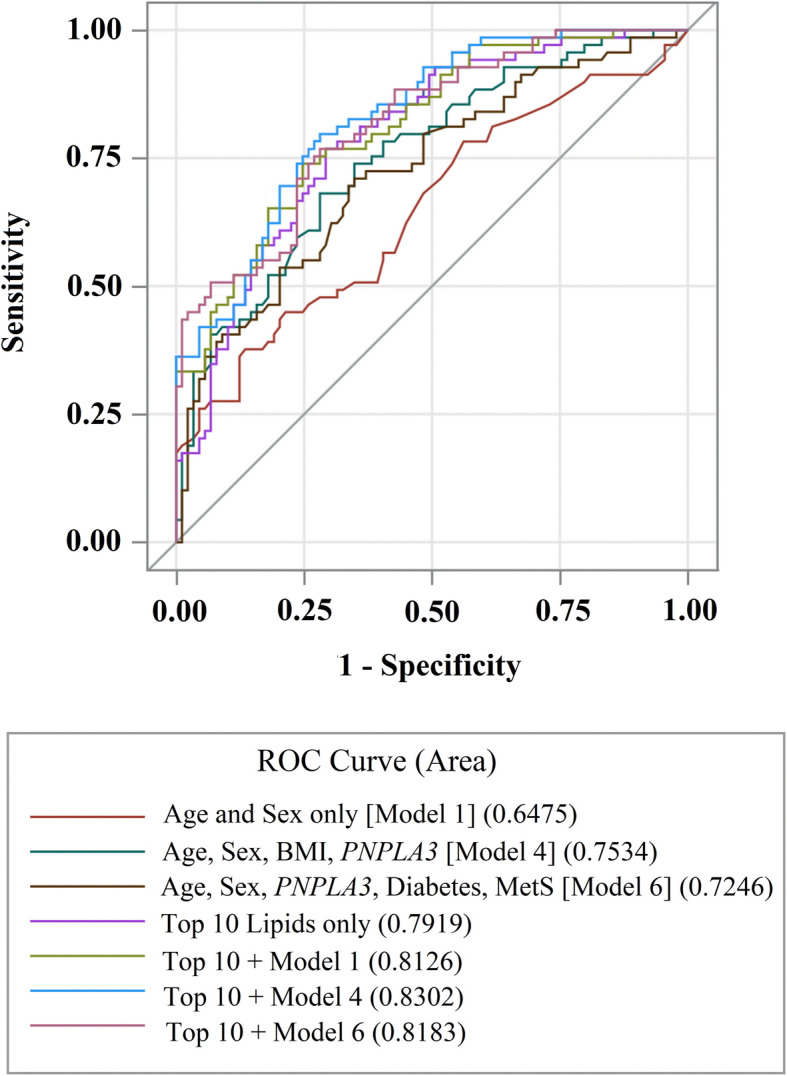
Receiver Operator Characteristic (ROC) curves of the top 10 lipids and other models Sensitivity (ability of panel to correctly identify NAFLD cases) and 1-specificity (ability of test to correctly identify healthy controls) are plotted. Area under the ROC curve (AUROC) is the predictive power of the panel to differentiate cases from controls. Perfect prediction would result in an AUROC of 1.0; random chance corresponds with an AUROC of 0.50. The top ten lipid species alone, as determined from the random forest analysis, are better predictors of NAFLD than models that include age, sex, BMI, *PNPLA3* genotype, diabetes, and/or metabolic syndrome (MetS). Abbreviations: *AUROC* area under the ROC curve, *BMI* body mass index *MetS* metabolic syndrome, *NAFLD* nonalcoholic fatty liver disease, *ROC* Receiver Operator Characteristic

An AUROC analysis was conducted to determine if a combination of the 10 most important lipids can predict case versus control status accurately, compared to other models (Fig. 5). Model 1, which only included age and sex, was able to predict case/control status 65% of the time (95% CI = 56–74%). A model that included age, sex, BMI, and *PNPLA3* genotype was able to estimate case/control status 75% of the time (95% CI = 67.8–82.9%). Model 6, which included age, sex, *PNPLA3* genotype, diabetes and metabolic syndrome was able to estimate case/control status 72% of the time (95% CI = 64–80%). A model that only included the top 10 lipids predicted case/control status accurately 79% of the time (95% CI = 72–86%), which is higher than the aforementioned models. Adding other covariates, including age, sex, *PNPLA3* genotype, diabetes, and metabolic syndrome to the panel of lipids further improved prediction to between 81% (95% CI = 75–88%) and 83% (95% CI = 77–89%) (Fig. 5). All three models that included the top 10 lipids plus other covariates were significantly more accurate at predicting case/control status than Model 6 (*P* < 0.05). Together, these data suggest that the circulating lipidome of Mexicans with NAFLD can have distinct features, which may be used to better discriminate disease.

## Discussion

This study identified differences in the composition of specific lipid species and subclasses in a sample of NAFLD cases and healthy controls from Mexico, where 75.2% of the population over age 20 is overweight (39.1%) or obese (36.1%) [64], and the prevalence of NAFLD among adults age 20 years or older is estimated to be over 50% [65]. Thus, this is a high-risk, yet understudied population. Another objective was to investigate whether any lipid species or a combination of specific lipids could be used to distinguish NAFLD cases from controls. To accomplish these goals, the clinical data and serum biospecimens from Mexican adults who are participating in a longitudinal study were examined. The findings of this study contribute to the lack of published research that has examined the association between serum lipids and NAFLD in Latino adults. For example, lipidomics analyses were conducted on plasma from Mexican American participants in the San Antonio Family Heart Study to investigate links with cardiovascular disease [65] and hypertension [66], but they did not examine the association between NAFLD and specific lipids. Another study examined 39 obese, Latino adolescents aged 11–17 years, of which 30 were determined to have NAFLD using magnetic resonance spectroscopy. Pathway analysis revealed that several lipid metabolism and amino acid pathways were dysregulated in the NAFLD cases. These include tyrosine metabolism, which was the most affected, as well as fatty acid activation, and branched-chain amino acid (BCAA) degradation [67]. However, there are no other published studies that have explored serum lipid signatures that are specific to NAFLD cases in adult Latino populations.

The analysis of neutral lipids conducted as part of this study indicates that the amounts of TAGs in NAFLD samples was higher than in controls, while the LPC pool was observed to be lower on average in the NAFLD serum samples. The pool size of CE was also lower in NAFLD cases compared to controls, after adjusting for covariates such as age, sex, BMI, diabetes, metabolic syndrome status, and *PNPLA3* genotype. The LPC and SM pools were also decreased even after adjusting for age, sex and *PNPLA3* genotype, however, after additionally adjusting for BMI, diabetes and metabolic syndrome, this significance was lost. TAG amounts were no longer significant when adjusting for any of the covariates, indicating that other factors, rather than NAFLD, are driving the observed changes in the size of the TAG pool.

The results of the individual lipid analyses are consistent with those of other studies [25, 42–44, 46, 47, 66, 67], as well as for specific lipids, such as CEs (15:0), (17:0), (18:1), (20:1), (20:2), (22:4) [66] and (22:6) [43], and LPCs (18:1) and (18:2) [42]. Controlling for variables other than age and sex led to a drop in the number of significant lipid metabolites, particularly TAGs, especially after adjusting for diabetes and metabolic syndrome status. This is expected due to the overlapping nature of metabolic diseases with triglyceride levels [68]. Another study that examined the liver lipidome in liver biopsies found that an increase in liver fat and NASH was associated with CER-enriched liver lipidome in patients with “Metabolic NAFLD”, compared to those with “*PNPLA3* NAFLD [69].” However, the results of this study did not uncover a strong association between CER and NAFLD. The findings from the random forest analysis indicate that LPC(17:0) is one of the most important determinants or predictor lipids to differentiate NAFLD cases and healthy controls. This is in contrast to recent findings that have shown a negative association between circulating odd chain fatty acids, in particular C15:0 and C17:0, with metabolic disease risks [70, 71]. PCs, and LPCs, by extension, were also negatively associated with NAFLD in a study of obese patients undergoing bariatric surgery in the Netherlands [66]. Several CEs, TAGs, and PCs were also commonly in the top 20.

Current clinical lipid panels are limited in their scope, usually measuring total cholesterol, triglycerides and lipoprotein amounts in the blood of individuals. To date, these limited lipid metabolic parameters have not yielded clinically actionable information regarding NAFLD diagnosis, prognosis and disease management. The results of this pilot study suggest that developing a clinical test that measures a broader panel of lipid subclass pool sizes could be useful in the diagnosis and potential management of NAFLD. Monitoring the amounts of these lipid subclasses could also provide insights as to whether an individual will progress to more severe forms of liver disease such as NASH and cirrhosis.

Of note, the AUROC analysis indicates that no single lipid predicted case/control status better than 71% accuracy after adjusting for age, sex, *PNPLA3* genotype, diabetes and metabolic syndrome. Including the top 10 lipids improved accuracy to almost 80%, which is better than a model with age, sex, BMI and *PNPLA3* genotype (75%). When combined, and after adjusting for age, sex, BMI, and *PNPLA3* genotype, the top 10 lipids we identified predicted case/control status better than an AUROC of 0.83, and was significantly better than just the top 10 lipids alone (*p* < 0.05). These findings suggest that a panel of individual lipids may be a viable option as a clinical indicator of NAFLD. However, the additional covariates included in the panel were not determined using model selection techniques and these preliminary results need to be validated in a large sample of NAFLD cases and controls.

Although the results of this study indicate that TAG amounts were not specifically associated with NAFLD after adjusting for covariates, measuring the desaturation state of TAGs provided useful information. By applying an algorithm that determines the utilization of saturated, monounsaturated and polyunsaturated acyl tails in the TAG pool, the NAFLD cases in this cohort were observed to have an enrichment for TAGs that were highly saturated (i.e. containing little or no polyunsaturated acyl tails). It has been shown that saturated FFAs are associated with a greater lipotoxicity in NAFLD when compared to monounsaturated FFAs [72, 73]. Another study conducted among HCC patients found that the more serious the disease, the higher the saturated TAG concentration in affected tissues [74]. They also found that TAGs with more than 2 double bonds in particular were down-regulated in HCC [74]. Thus, it may be of value to specifically monitor the desaturation state of the TAG pool to facilitate diagnosis and progression of NAFLD.

### Strengths and limitations

This is the first lipidomics study among Latino adults with NAFLD, an understudied, high-risk group. The findings of this study confirm the results of previous studies in other populations [25, 42–44, 46, 47, 66] and provide novel results that could inform the clinical management of NAFLD patients. However, due to limitations in sample size, these findings will need to be replicated in larger cohorts and future investigations. This study has some limitations. First, the Health Worker Cohort Study (HWCS) participants are mostly women (75%) who have a higher education level and overall better health than the general population of Mexico. The HWCS participants are primarily middle-income, which represents an estimated 34% of the population. Although the sample is not generalizable to the Mexican population, the internal validity of the study is unlikely to be compromised. Second, the study was conducted with Mexicans and does not include other Latino groups. Therefore, these results might not be representative of other Latino groups due to the heterogeneity of health status among Latinos. Third, certain analyses (e.g. sex-, menopause, or *PNPLA3* status-stratified analyses) could not be performed due to sample size limitations, which affected power. Fourth, although histology is considered the “gold standard” to diagnose and stage NAFLD, the NAFLD cases included in this analysis were clinically confirmed using elevated liver enzyme tests and ultrasound of the liver, after excluding alcohol abuse. As a result, these findings should be interpreted with caution since ultrasonography is unreliable at detecting smaller amounts of fat in the liver [3], and cannot detect inflammation or fibrosis, which can indicate more advanced NAFLD. There might also be misclassification bias if some participants reported that they were not drinkers when in fact they were. Additionally, we were unable to assess whether participants had NASH and therefore, could not identify lipids which may be unique to NASH patients, to differentiate from both normal controls as well as NAFL. In several studies, some lipid concentrations were only significantly different between NASH and healthy controls or NASH vs NAFL, while others found that the concentration difference between NAFL vs NASH was opposite of that for NAFL vs controls [25, 42, 43, 73, 75]. Future analyses could include per unit-concentration differences in odds of NAFLD, testing both linear and non-linear relationships between lipid species and NAFLD (e.g. quadratic, cubic, etc.). Finally, although homogenization of tissue results in dilution and averaging of molecules, and serum lipids do not exclusively reflect liver lipids [76–79].

Other areas of future research should examine why highly saturated TAGs are enriched in Latino NAFLD patients. A study that investigated if a sub-set of TAGs were associated with hepatic steatosis found that TAGs containing saturated and mono-saturated FFAs with 16–18 carbons were related to a greater intake of carbohydrates and saturated fat [80]. Another study examined how dietary macronutrient composition influences intrahepatic triglyceride content and found that an increase in carbohydrate consumption increases hepatic de novo lipogenesis, while saturated fat induced the greatest increase in intrahepatic triglyceride content [81]. Future studies should also explore the association between the *PNPLA3*-I148M variant and lipid composition among Latino patients with NAFLD. Studies with non-Latino populations have found that the *PNPLA3*-I148M variant increases hepatic retention of polyunsaturated fatty acids [82] and polyunsaturated triglycerides in human adipose tissue [83]. Since Latinos have the highest prevalence of the *PNPLA3*-I148M variant [34–36] and the highest rates of NAFLD in the U.S. [6–10], there should be more research studies that focus on this high-risk, understudied population.

## Conclusion

This study examined lipidomics, genetic, and clinical data to investigate the association between specific lipids and risk of NAFLD in a sample of adults from Mexico. Significant differences were identified in the prevalence of certain lipids and lipid features between the NAFLD cases and the healthy controls. A panel of 10 lipids was generated that, in tandem with other covariates, distinguished NAFLD cases from controls with an AUROC of 83%. These findings suggest that a targeted panel of specific lipids and other covariates could be used to detect NAFLD, and provide clinically relevant information regarding progression and response to treatments. Currently, there are no FDA-approved medications to treat NAFLD or NASH, but several drugs are expected to become available in the next few years. Thus, it is becoming increasingly relevant to identify non-invasive ways to diagnose NAFLD patients, so they can be targeted for treatment. Although additional studies are needed to validate these findings in Latinos and other populations, the results of this study point to a better understanding of how lipid dysregulation may contribute to the increased liver disease susceptibility observed among Mexicans. This study also contributes to the identification of potential lipid biomarkers that may help to improve the detection and treatment of NAFLD.

## Supplementary Information

**Additional file 1.** Pearson’s linear correlation coefficients between all lipid species. Values reported are correlation coefficients, which lie in the range [− 1,1]. Perfect positive correlation corresponds to a value of 1, as seen by the diagonal red line where each lipid is correlated with itself. A perfect negative correlation corresponds to a value of − 1. No correlation corresponds to a value of 0. NA values are replaced with 0. Abbreviations: *CE* cholesterol ester, *CER* ceramide, *DAG* diacyglycerol, *FFA* free fatty acid, *HCER* hexosylceramide, *LCER* lactosylceramide, *LPC* lysophosphatidylcholine, *LPE* lysophosphatidylethanolamine, *PC* phosphatidylcholine, *SM* sphingomyelin, *TAG* triacylglycerol.

**Additional file 2.** Heat map of all lipids measured in serum from nonalcoholic fatty liver disease (NAFLD) cases and controls. Rows are centered; unit variance scaling is applied to rows, displayed as colors ranging from red to blue as shown in the key (Color range: − 3.2 to 3.2). Missing values shown in white.

**Additional file 3 **Heat Map of all (a) CE, (b) LPC and (c) TAG lipids measured in serum from NAFLD cases and controls. Rows are centered; unit variance scaling is applied to rows, displayed as colors ranging from red to blue as shown in the key. Color ranges are as follows: CE: − 2.58 to 2.58, LPC: − 3.29 to 3.29, TAG: − 1.58 to 1.58. Missing values shown in white. Abbreviations: *CE* cholesterol ester, *LPC* lysohphosphatidylcholine, *NAFLD* nonalcoholic fatty liver disease, *TAG* triacylglycerol.

**Additional file 4.** AUROC of top 20 lipids as determined by random forest analysis.

**Additional file 5.** Difference in top 20 lipids between NAFLD cases and controls. The median and 25–75% interquartile range (IQR) are presented in box plots with whiskers representing 1.5x the IQR bounded by the highest and lowest samples. *P* values indicated above comparisons of case and control (two-tailed unpaired Student’s t test). * *P* < 0.05, **P < 0.01; ***P < 0.001 Abbreviations: *CE* cholesterol ester, *CER* ceramide, *DAG* diacylglycerol, *DCER* dihydroceramide, *FFA* free fatty acid, *HCER* hexosylceramide, *LCER* lactosylceramide, *LPC* lysophosphatidylcholine, *LPE* lysophosphatidylethanolamine, *PC* phosphatidylcholine, *SM* sphingomyelin, *TAG* triacylglycerol.

## Data Availability

Post Processed lipid species concentration data is in Supplemental Lipidomics File 1. Lipidomics data will be made available at the NIH Common Fund’s National Metabolomics Data Repository website, the Metabolomics Workbench (https://www.metabolomicsworkbench.org) upon acceptance of the manuscript.

## Abbreviations

ALT: Alanine aminotransferase
ANCOVA: Analysis of covariance
AST: Aspartate aminotransferase
AUROC: Area under the receiver operating characteristic curve
BMI: Body mass index
BCAA: branched-chain amino acid
CE: Cholesterol ester
CER: Ceramide
CI: Confidence interval
DAG: Diacyglycerol
DCER: Dihydroceramide
FDR: False discovery rate
FFA: Free fatty acid
HWCS: Health Worker Cohort Study
HCC: Hepatocellular carcinoma
HCER: Hexosylceramide
HDL-C: High-density lipoprotein
IQR: Interquartile range
IU/L: International units per litre
LCER: Lactosylceramide
LDL-C: Low-density lipoprotein cholesterol
LPE: Lysophosphatidylethanolamine
LPC: Lysophosphatidylcholine
MRM: Multiple-reaction monitoring
NAFL: Nonalcoholic fatty liver
NAFLD: Nonalcoholic fatty liver disease
NASH: Nonalcoholic steatohepatitis
PC: Phosphatidylcholine
PE: Phosphatidylethanolamine
ROC: Receiver operating characteristic
SNP: Single nucleotide polymorphisms
SM: Sphingomyelin
SD: Standard deviation
TAG: Triacylglycerol.
