# Supplementary figures and images for: Serum lipids are associated with nonalcoholic fatty liver disease: a pilot case-control study in Mexico
Source: Lipids Health Dis. 2021 Oct 10;20:136. doi: 10.1186/s12944-021-01526-5 (PMC8504048; doi:10.1186/s12944-021-01526-5)

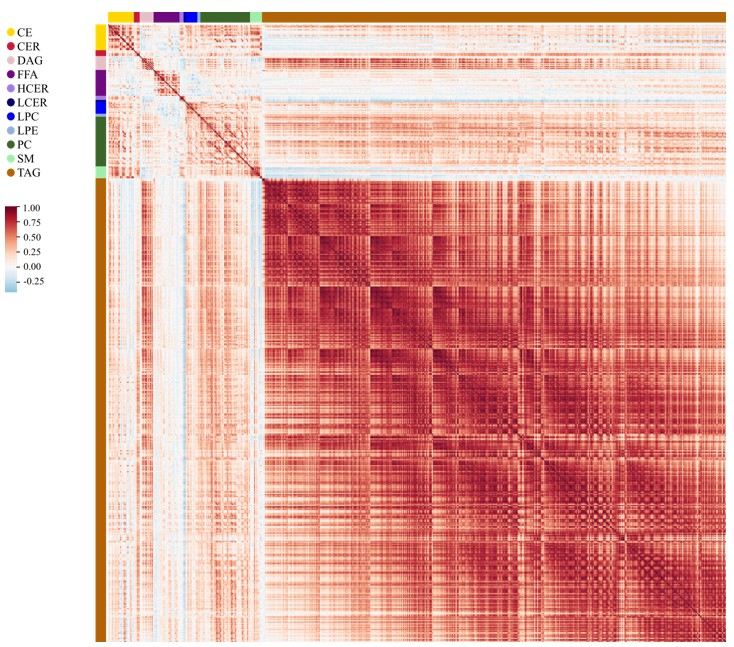

Supplement: Supplementary file 1 — Additional file 1. Pearson’s linear correlation coefficients between all lipid species. Values reported are correlation coefficients, which lie in the range [− 1,1]. Perfect positive correlation corresponds to a value of 1, as seen by the diagonal red line where each lipid is correlated with itself. A perfect negative correlation corresponds to a value of − 1. No correlation corresponds to a value of 0. NA values are replaced with 0. Abbreviations: CE cholesterol ester, CER ceramide, DAG diacyglycerol, FFA free fatty acid, HCER hexosylceramide, LCER lactosylceramide, LPC lysophosphatidylcholine, LPE lysophosphatidylethanolamine, PC phosphatidylcholine, SM sphingomyelin, TAG triacylglycerol. [file 12944_2021_1526_MOESM1_ESM.jpg]

**
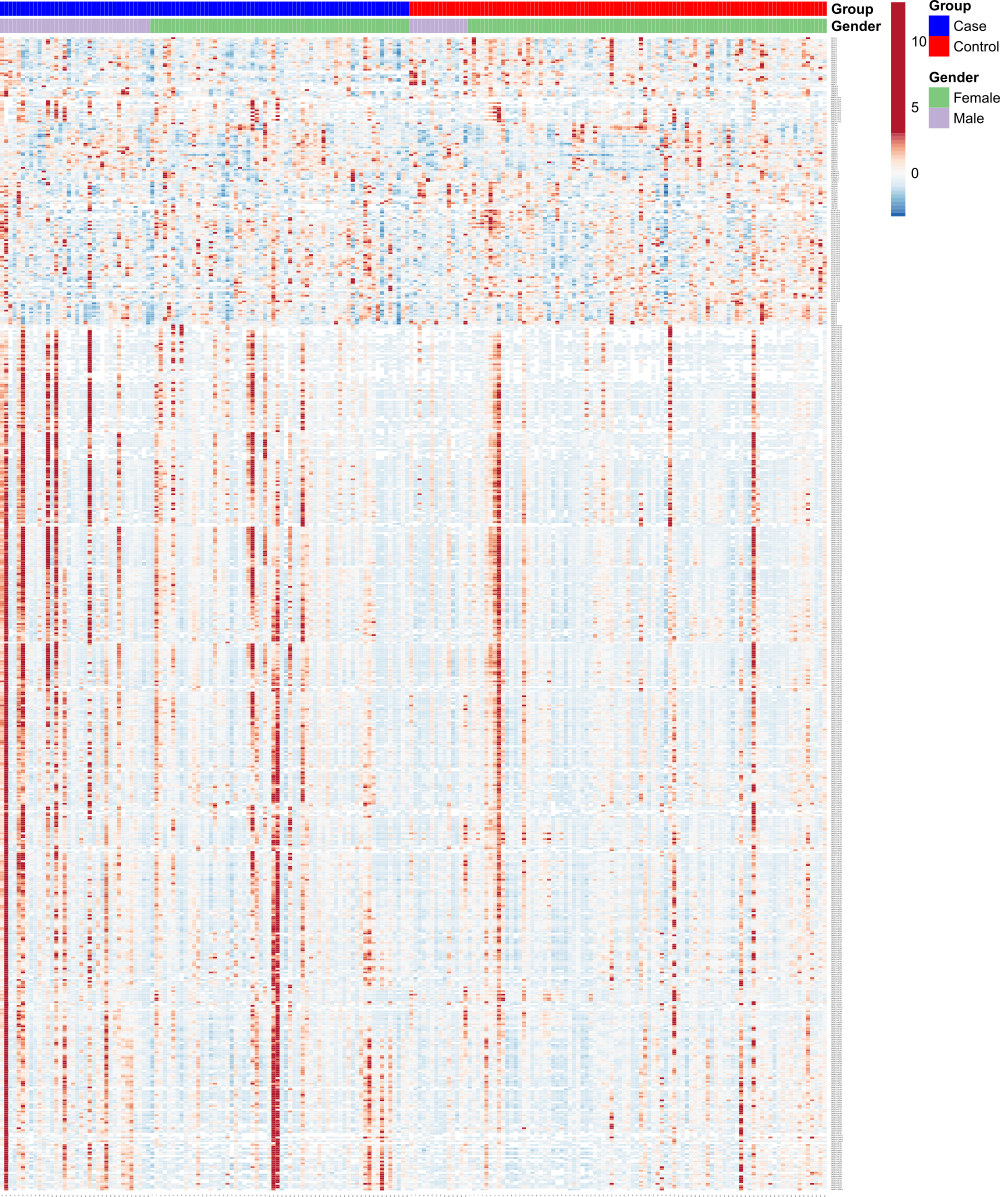
**

Supplement: Supplementary file 2 — Additional file 2. Heat map of all lipids measured in serum from nonalcoholic fatty liver disease (NAFLD) cases and controls. Rows are centered; unit variance scaling is applied to rows, displayed as colors ranging from red to blue as shown in the key (Color range: − 3.2 to 3.2). Missing values shown in white. [file 12944_2021_1526_MOESM2_ESM.docx]

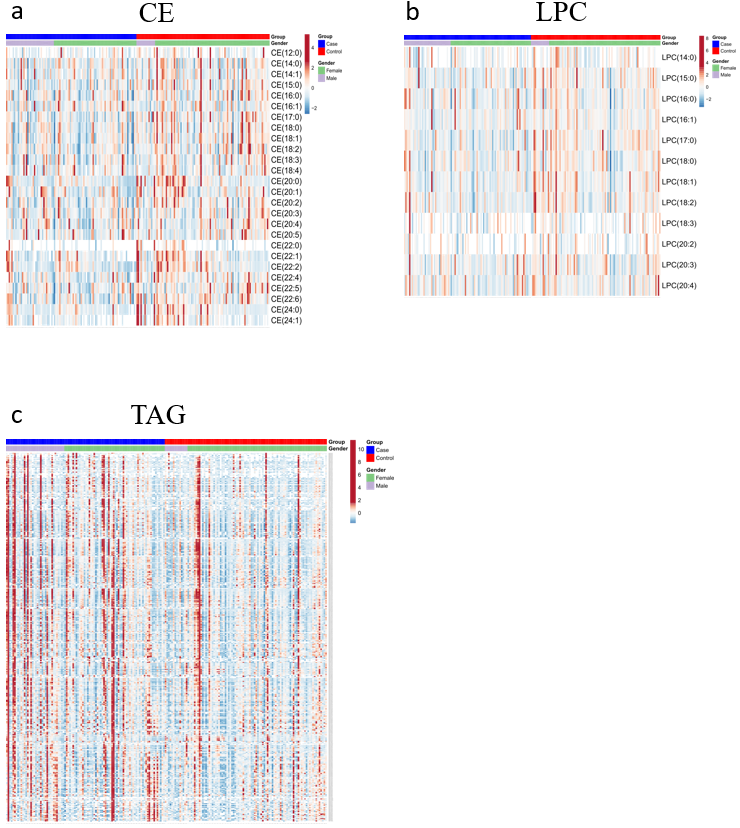

Supplement: Supplementary file 3 — Additional file 3 Heat Map of all (a) CE, (b) LPC and (c) TAG lipids measured in serum from NAFLD cases and controls. Rows are centered; unit variance scaling is applied to rows, displayed as colors ranging from red to blue as shown in the key. Color ranges are as follows: CE: − 2.58 to 2.58, LPC: − 3.29 to 3.29, TAG: − 1.58 to 1.58. Missing values shown in white. Abbreviations: CE cholesterol ester, LPC lysohphosphatidylcholine, NAFLD nonalcoholic fatty liver disease, TAG triacylglycerol. [file 12944_2021_1526_MOESM3_ESM.docx]

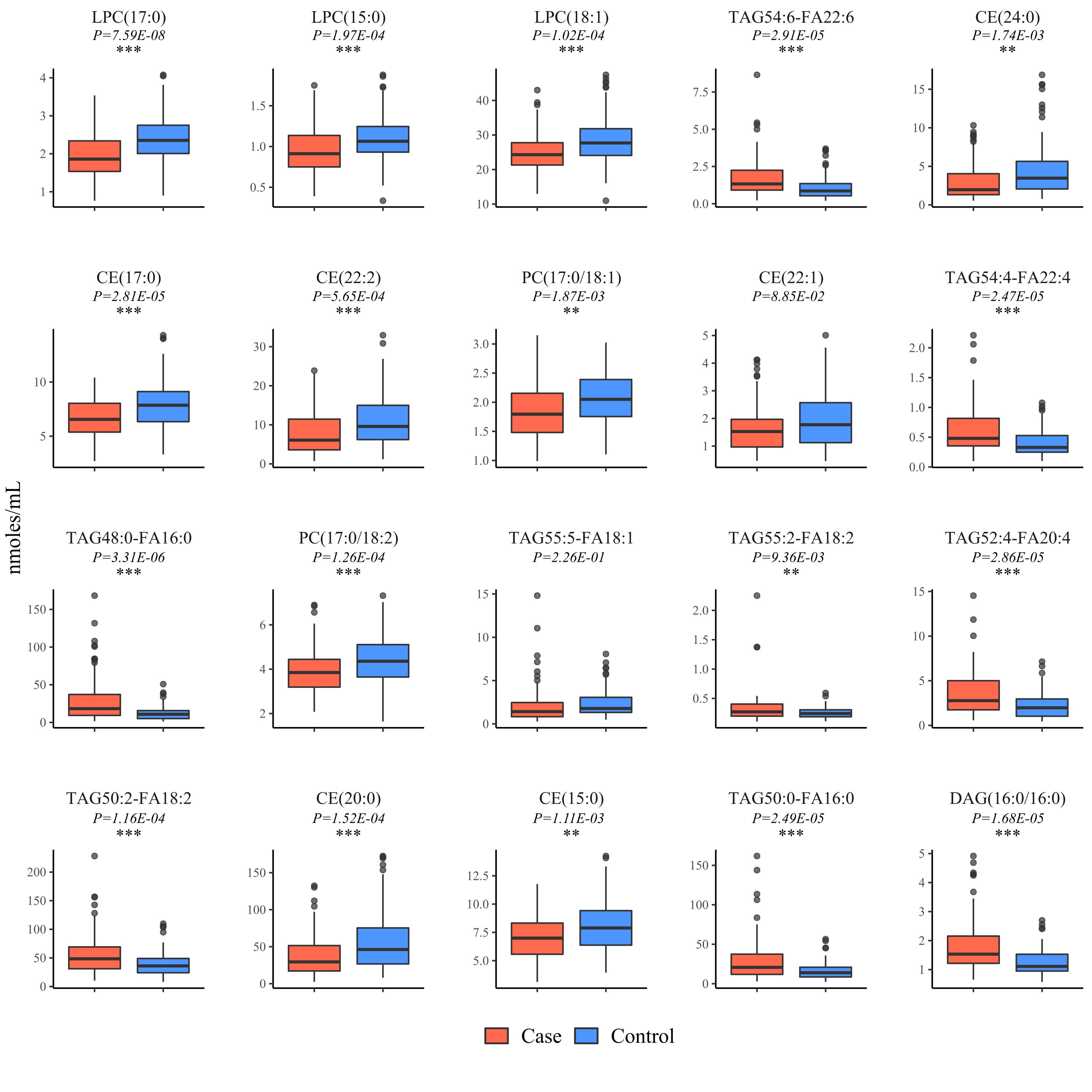

Supplement: Supplementary file 5 — Additional file 5. Difference in top 20 lipids between NAFLD cases and controls. The median and 25–75% interquartile range (IQR) are presented in box plots with whiskers representing 1.5x the IQR bounded by the highest and lowest samples. P values indicated above comparisons of case and control (two-tailed unpaired Student’s t test). * P < 0.05, **P < 0.01; ***P < 0.001 Abbreviations: CE cholesterol ester, CER ceramide, DAG diacylglycerol, DCER dihydroceramide, FFA free fatty acid, HCER hexosylceramide, LCER lactosylceramide, LPC lysophosphatidylcholine, LPE lysophosphatidylethanolamine, PC phosphatidylcholine, SM sphingomyelin, TAG triacylglycerol. [file 12944_2021_1526_MOESM5_ESM.png]
