# Supplementary material for: Serum lipids are associated with nonalcoholic fatty liver disease: a pilot case-control study in Mexico
Source: Lipids Health Dis. 2021 Oct 10;20:136. doi: 10.1186/s12944-021-01526-5 (PMC8504048; doi:10.1186/s12944-021-01526-5)
Supplement: Supplementary file 4 — Additional file 4. AUROC of top 20 lipids as determined by random forest analysis. [file 12944_2021_1526_MOESM4_ESM.docx]

| **Lipid** | **AUROC^a^** |
| --- | --- |
| LPC(17:0) | 0.6769 |
| LPC(15:0) | 0.6741 |
| LPC(18:1) | 0.7082 |
| TAG54:6-FA22:6 | 0.6636 |
| CE(24:0) | 0.5353 |
| CE(17:0) | 0.5820 |
| CE(22:2) | 0.5406 |
| PC(17:0/18:1) | 0.6457 |
| CE(22:1) | 0.5406 |
| TAG54:4-FA22:4 | 0.6851 |
| TAG48:0-FA16:0 | 0.6814 |
| PC(17:0/18:2) | 0.6218 |
| TAG55:5-FA18:1 | 0.5467 |
| TAG55:2-FA18:2 | 0.5197 |
| TAG52:4-FA20:4 | 0.6818 |
| TAG50:2-FA18:2 | 0.6619 |
| CE(20:0) | 0.5588 |
| CE(15:0) | 0.5499 |
| TAG50:0-FA16:0 | 0.6875 |
| DAG(16:0/16:0) | 0.7090 |

**Additional file 4. AUROC of top 20 lipids as determined by random forest analysis**

^a^ Model adjusted for age, sex, *PNPLA3* genotype, diabetes, and metabolic syndrome status Abbreviations: *AUROC* area under the receiver operative characteristic curve, *LPC* lysohphosphatidylcholine, *TAG* triacylglycerol, *CE* cholesterol ester, *PC* phosphatidylcholine, *DAG* diacylglycerol
